# Opportunities and Alternatives of Modern Radiation Oncology and Surgery for the Management of Resectable Brain Metastases

**DOI:** 10.3390/cancers15143670

**Published:** 2023-07-19

**Authors:** Christian D. Diehl, Frank A. Giordano, Anca-L. Grosu, Sebastian Ille, Klaus-Henning Kahl, Julia Onken, Stefan Rieken, Gustavo R. Sarria, Ehab Shiban, Arthur Wagner, Jürgen Beck, Stefanie Brehmer, Oliver Ganslandt, Motaz Hamed, Bernhard Meyer, Marc Münter, Andreas Raabe, Veit Rohde, Karl Schaller, Daniela Schilling, Matthias Schneider, Elena Sperk, Claudius Thomé, Peter Vajkoczy, Hartmut Vatter, Stephanie E. Combs

**Affiliations:** 1 Department of Radiation Oncology, Technical University of Munich (TUM), Klinikum rechts der Isar, 81675 München, Germany; 2 Institute of Radiation Medicine (IRM), Helmholtz Zentrum München, 85764 Neuherberg, Germany; 3 Deutsches Konsortium für Translationale Krebsforschung (DKTK), Partner Site Munich, 80336 München, Germany; 4 Department of Radiation Oncology, University Medical Center Mannheim, Medical Faculty Mannheim, Heidelberg University, 68167 Mannheim, Germany; 5 Department of Radiation Oncology, University Medical Center, Medical Faculty, 79106 Freiburg, Germany; 6 Department of Neurosurgery, Faculty of Medicine, Technical University of Munich, 81675 München, Germany; 7 Department of Radiation Oncology, University Medical Center Augsburg, 86156 Augsburg, Germany; 8 Department of Neurosurgery, Charité-Universitätsmedizin Berlin, Corporate Member of Freie Universität Berlin, Humboldt-Universität zu Berlin, 10117 Berlin, Germany; 9 Berlin Institute of Health, Charité-Universitätsmedizin Berlin, 10117 Berlin, Germany; 10 German Cancer Consortium (DKTK), Partner Site Berlin, German Cancer Research Center (DKFZ), 69120 Heidelberg, Germany; 11 Department of Radiotherapy and Radiation Oncology, University Medical Center Göttingen, 37075 Göttingen, Germany; 12 Comprehensive Cancer Center Niedersachsen (CCC-N), 37075 Göttingen, Germany; 13 Department of Radiation Oncology, University Hospital Bonn, University of Bonn, 53127 Bonn, Germany; 14 Department of Neurosurgery, University Medical Center Augsburg, 86156 Augsburg, Germany; 15 Department of Neurosurgery, University Hospital Freiburg, 79106 Freiburg, Germany; 16 Department of Neurosurgery, University Medical Center Mannheim, Medical Faculty Mannheim, Heidelberg University, 68167 Mannheim, Germany; 17 Neurosurgical Clinic, Klinikum Stuttgart, 70174 Stuttgart, Germany; 18 Department of Neurosurgery, University Hospital Bonn, 53127 Bonn, Germany; 19 Department of Radiation Oncology, Klinikum Stuttgart Katharinenhospital, 70174 Stuttgart, Germany; 20 Department of Neurosurgery, Inselspital, Bern University Hospital, University of Bern, 3010 Bern, Switzerland; 21 Department of Neurosurgery, Universitätsmedizin Göttingen, 37075 Göttingen, Germany; 22 Department of Neurosurgery, University of Geneva Medical Center & Faculty of Medicine, 1211 Geneva, Switzerland; 23 Mannheim Cancer Center, University Medical Center Mannheim, Medical Faculty Mannheim, Heidelberg University, 68167 Mannheim, Germany; 24 Department of Neurosurgery, Medical University of Innsbruck, 6020 Innsbruck, Austria

**Keywords:** brain metastases, microsurgical resection, stereotactic radiation therapy, intraoperative radiation therapy, radiosurgery, neurosurgery, whole brain radiation therapy

## Abstract

**Simple Summary:**

Brain metastases are of increasing concern in the management of cancer patients. New systemic treatment options like specific targeting agents or immune checkpoint inhibitors allow for longer survival with a higher incidence of brain metastases over the course of the disease. Single or a few small, asymptomatic lesions are mainly treated with focal stereotactic radiotherapy. On the other hand, in many cases, large and, in particular, symptomatic metastases require microsurgical resection for immediate symptom relief. However, the indication for surgery can be challenging concerning the clinic, patient and tumor characteristics, and the availability of alternative conservative treatment options. Of note, with surgery alone, local recurrence rates are about 50%; therefore, additional RT is needed to improve outcomes.

**Abstract:**

Postsurgical radiotherapy (RT) has been early proven to prevent local tumor recurrence, initially performed with whole brain RT (WBRT). Subsequent to disadvantageous cognitive sequalae for the patient and the broad distribution of modern linear accelerators, focal irradiation of the tumor has omitted WBRT in most cases. In many studies, the effectiveness of local RT of the resection cavity, either as single-fraction stereotactic radiosurgery (SRS) or hypo-fractionated stereotactic RT (hFSRT), has been demonstrated to be effective and safe. However, whereas prospective high-level incidence is still lacking on which dose and fractionation scheme is the best choice for the patient, further ablative techniques have come into play. Neoadjuvant SRS (N-SRS) prior to resection combines straightforward target delineation with an accelerated post-surgical phase, allowing an earlier start of systemic treatment or rehabilitation as indicated. In addition, low-energy intraoperative RT (IORT) on the surgical bed has been introduced as another alternative to external beam RT, offering sterilization of the cavity surface with steep dose gradients towards the healthy brain. This consensus paper summarizes current local treatment strategies for resectable brain metastases regarding available data and patient-centered decision-making.

## 1. Introduction

The treatment of brain metastases (BM) is of increasing interest in the management of cancer patients. About 10–40% of them develop BM at the different stages of the disease, and it is not unusual that symptomatic lesions lead to the first diagnosis of cancer [1,2]. The incidence of BM is tenfold higher than that for gliomas, accounting for 50% of all intracranial neoplasia [3].

Regarding single primary entities, the risk of having intracranial tumor involvement over the clinical course is 37% for malignant melanoma, 50% for non-small-cell lung cancer (NSCLC), >50% for metastatic human epidermal growth factor receptor-positive (HER+) breast cancer or triple-negative breast cancer (TNBC), and 80% for small-cell lung cancer (SCLC) [4,5,6,7]. The most frequent primaries are SCLC or NSCLC, breast cancer, malignant melanoma, renal cell carcinoma (RCC), and colorectal cancer (CRC); the former three primaries constitute 80% due to the high incidences [8,9]. Therefore, BM screening with cranial magnetic resonance imaging (MRI) is recommended in the primary staging of SCLC/NSCLC or higher-stage malignant melanoma, but not yet for other high-risk entities such as TNBC [2]. In childhood and adolescence, BMs are rare; in most cases, the primary tumors are neuroblastoma, sarcoma, nephroblastoma, melanoma, and germline tumors [10,11]. All brain regions can be affected, with three-quarters showing parenchymal growth. Out of this, 80% are located supra-tentorial, 15% in the cerebellum, and 5% in the brain stem [12,13]. BM can often be detected posterior to the Sylvian fissure at the junction of the temporal, parietal, and occipital lobes, representing the supply zone of the median cerebral artery [13]. BM are singular/solitary in 72% of cases; SCLC/NSCLC and melanoma show more often multiple lesions compared to RCC and CRC [14,15]. Overall, the incidence of BM is increasing because of better systemic treatment options, leading to improved survival for many cancer types and hence a higher risk for BM over the course of the disease [16]. These new drugs consist of small molecules and antibodies targeting tyrosine kinases, molecular alterations such as epidermal growth factor receptor (EGFR) mutations or anaplastic lymphoma-kinase (ALK) rearrangements, or immune checkpoint inhibitors, which contrary to classic chemotherapies, can better cross the blood–brain barrier for intrathecal anti-tumor effects and therefore protracted development of BM [17,18,19,20]. BM screening in NSCLC/SCLC, or melanoma patients often detects small and clinically unapparent BM that can be easily treated with definitive stereotactic radiosurgery (SRS). But not all BMs are detected at an early stage where they are amenable to radiotherapy (RT). Large or symptomatic lesions in eloquent brain areas can cause disability and even life-threatening conditions that require rapid decompression for fast and persistent symptom relief [21,22]. Furthermore, resection supports the histopathologic diagnosis in patients with cancer of unknown primary or when changes in molecular profiles compared to the primary tumor are suspected [23,24]. However, local recurrence rates after the resection of a single BM are up to 50% [25,26,27]. Therefore, RT is indicated to improve local control (LC) at the site of resection. Up to date, several techniques are available, such as whole brain radiation therapy (WBRT), local SRS or hypo-fractionated stereotactic RT (hFSRT) to the cavity, neo-adjuvant stereotactic radiosurgery (N-SRS), and low-energy X-ray intraoperative RT (IORT).

In this report, we highlight the role of surgery and discuss the approaches of neoadjuvant, intraoperative, and adjuvant RT in the clinical management of BM.

## 2. Surgery

The indications for BM surgery have recently been updated in the ASCO-SNO-ASTRO guideline and the EANO-ESMO clinical practice guidelines [23,24]. BMs affect an exceptionally high number of cancer patients and thereby represent a common challenge in medical care for the population [28,29]. In 20 to 25% of cases, a BM may be the primary cause of the diagnosis of an underlying cancerous disease [28,30,31]. Due to significant improvements in cancer therapies and imaging techniques as well as an aging population, a growing number of patients are eligible for and in need of neurosurgical treatment of brain tumors, a very heterogeneous group of which BMs represent the most prevalent subgroup by a large margin. The antique assumption that BMs are easily removed due to being well-circumscribed and having non-invasive growth has been long disproven [32,33,34]. BM of SCLC or melanoma can infiltrate the brain by several millimeters [35]. This might contribute to the lower-than-expected complete resection rates and higher local recurrence rates after surgery for BMs [34]. Being diagnosed with a BM entails drastic consequences for patients’ quality of life (QoL), functional autonomy, and socioeconomic status. In addition to being diagnosed with cancer of any origin, BMs are often viewed as terminal and incurable by patients, which heavily impedes their functional autonomy and well-being. It must be emphasized that cerebral metastases may produce a range of neurological symptoms leading to severe disability and impairment of QoL [30,31,36].

The resection of BMs is generally not of a curative nature. As the presence of a BM merely mirrors the progression of the causative systemic disease, any cranial surgery is only indicated for symptom control and securing a histopathological diagnosis in cases of a yet unknown primary cancer [34]. While the currently available evidence does not support neurosurgical resection as a curative intervention, complete resection has recently been shown to play a significant role in survival extension in addition to being a measure of symptom control and preservation of QoL [28,29,31,32,34,37,38].

### 2.1. Indications for the Resection of Asymptomatic Brain Metastases

Indications for surgery in asymptomatic BMs can be categorized based on clinical, oncological, and histopathological considerations. Patients with large tumors with mass effect, large lesions (>3 cm) in the posterior fossa, and a single BM per se must be considered for surgical resection. Moreover, patients suffering from necrotic or cystic BM as shown by MRI and responding less well to RT [39,40], might also be suitable for resection [23]. Here, radiomics can help evaluate the grade of response to SRS [41]. Additionally, on an interdisciplinary basis, the decision for the resection of asymptomatic BM leading to symptoms or obstructive hydrocephalus due to edema during RT should also be discussed regarding the option of prophylactic resection.

Surgery may also be considered in patients who require steroids and are eligible for immune checkpoint inhibition [24]. From an oncological perspective, (i) patients with tumors that rarely cause BMs, (ii) patients with unclear histology, (iii) patients with no primary tumor known, or (iv) patients with multiple primary tumors should be considered for surgery [23]. Tumors that might undergo changes in their molecular profile, such as breast cancer BM, may influence clinical decision-making and should therefore also be considered for surgery [42].

### 2.2. Indications for the Resection of Symptomatic Brain Metastases

Despite having no clear recommendation for surgery in patients with asymptomatic BMs in the latest ASCO-SNO-ASTRO guideline but for local therapy defined as “radiosurgery or radiation therapy and/or surgery”, their clinical interpretation concludes with the highlighting of benefits for patients with symptomatic BMs undergoing surgery [24]. Current EANO-ESMO clinical practice guidelines define specific scenarios for the consideration of surgery for its immediate therapeutic effect in patients with BMs larger than 3 cm that lead to elevated intracranial pressure (ICP) or neurological deficits when located in eloquent brain areas. These European guidelines clearly recommend the consideration of surgery in patients with acute symptoms of raised ICP [23]. Although randomized controlled trials showing the benefits of surgery were published more than 10 years ago [43,44], multiple non-randomized studies examined the current clinical standard to resect symptomatic BMs as a first step of comprehensive cancer therapy [45,46,47,48,49].

With this in mind, the definition of symptoms for the interdisciplinary and surgical decision-making process in the last step must be mentioned and additionally impacts the survival prognosis [50]: clinical motor, sensory, and language impairment, as well as a history of generalized seizures, are clear symptoms in patients with BM, leading to the indication for surgery. Moreover, in most cases with more than one BM, the symptomatic one can be clearly localized on MRI. The same applies to BM, leading to cerebrospinal fluid (CSF) circulation disorders and elevated ICP with related symptoms, especially in infratentorial locations. However, symptoms such as personality changes, reduced cognitive flexibility, visual disturbances, further higher cortical or subcortical functions, or symptoms related to focal seizures being more difficult to detect during clinical examination also must be considered. Therefore, the quality of clinical examinations and interdisciplinary discussions are highly important for the treatment of BM [51]. Furthermore, the perioperative use of the modern armamentarium of pre- and intra-operative techniques and tools for imaging and monitoring of the patients’ functional status is standardly required. Additional aspects such as the overall performance status of the patient, the current control of systemic disease, further diseases, and medication must be considered for this case-by-case decision-making process [24].

### 2.3. Role of Surgery in Multiple or Recurrent BMs

Recommendations for the role of surgery in patients with multiple BMs must be differentiated based on the parameters mentioned above. While patients with multiple BMs and/or uncontrolled systemic disease are less likely to benefit from surgery [24], surgical resection should be considered in eligible patients with multiple resectable BMs and should be focused on symptomatic lesions [23]. Even more, in cases of acute neurological deterioration due to elevated ICP based on CSF circulation disturbances (e.g., obstruction of the fourth ventricle), surgical options such as continuous CSF drainage with ventricular-perotoneal/-atrial shunting as a kind of best supportive care should at least be discussed with patients and relatives, even though systemic therapy as well as radiotherapeutic options are missing.

### 2.4. Re-Operation of Recurrent Brain Metastases and Radiogenic Changes

Due to the ongoing developments in oncology and especially with the introduction of targeted therapies, the overall survival of patients with metastatic disease has markedly increased. Therefore, the number of patients presenting with local recurrence following radiotherapy or following resection of BM is also rising [52,53,54]. Regarding the surgical therapy of recurrent BMs, several non-randomized studies have analyzed the impact of re-surgery in these patients [55,56,57,58,59].

There is some consensus that a progressive lesion in a patient with a controlled systemic disease who is presenting with a good functional condition (Karnofsky performance status (KPS) > 60) is a good candidate for surgical intervention [54,59,60,61,62,63]. The benefits of surgical resection are most pronounced in patients with symptomatic recurrent BMs because surgery will lead to improvements in functional outcome and prolonged functional independence in most cases [54,55,62]. Furthermore, a new evaluation of the molecular biology of the tumor may also lead to new systemic treatment options that may lead to more favorable oncological results [48]. The median overall survival (OS) following neurosurgical resection for recurrent BMs is highly dependent on the baseline functional status [64], the extent of resection [59], and the elapsed time between surgeries [55]. However, longer periods of progression-free survival (PFS) are probably due to a favorable response to the systemic treatment rather than a surgery-related factor. Thereby, OS is reported to range between 7 [59] and 20 months [54].

Because almost all patients with recurrent BMs present after having radiotherapy and/or chemotherapy, causing scarring and dural adhesions, there is an increased risk of wound healing disorder, infection, and CSF fistula following neurosurgical resection of recurrent BMs [62,65,66]. However, the reported morbidity in most of the surgical series reporting on initial [43,44,48,67] and recurrent surgery [54,55,59,60,64] for BMs does not show any significant differences. The reported higher morbidity in some selected series [66] may be more dependent on a specific patient cohort in a given study than on surgical factors. Similarly, the mortality rates following recurrent surgery for BM range between 0 and 3% [54,55,59,60,64] and do not differ from the rates following initial surgery [43,44,48,67].

In cases with recurrent BM that are not eligible for neurosurgical resection, some are advocating for laser interstitial thermal therapy (LITT) [68,69]. There is limited data suggesting that complete ablation yields favorable LC and improved QoL [70,71]. However, these potential benefits of LITT need to be weighed against potential devastating complications such as malignant brain edema and the development of new severe neurological deficits [70,71,72,73].

In summary, for a patient with a good functional status (KPS > 60) and a symptomatic recurrent BM, neurosurgical resection should be recommended in all cases where surgical removal is feasible. Surgical resection aims to improve symptoms, prolong the functional independence of the patients, and achieve a new evaluation of the molecular biology of the tumor. Recommendations for LITT for the treatment of recurrent BM cannot be given since data are still scarce and complication rates are relatively high.

With respect to the patient’s status, considering symptoms, systemic disease burden, overall performance status, and post-operative therapy options, the option for re-surgery must be evaluated again at the time of recurrent BM diagnosis, based on an interdisciplinary case-by-case discussion [23].

### 2.5. Surgical Techniques: Standard of Care and Emerging Technologies

Microscopic brain tumor resection is a highly standardized procedure and represents the gold standard in BM resection. The latest ASCO-SNO-ASTRO guidelines do not make recommendations regarding the differentiated type of resection, which can be en bloc or piecemeal [24]. For this differentiation of resection type, only one retrospective comparative cohort study could be identified analyzing the impact of the surgical methodology on the complication rate and clinical outcome of patients [74]. Additionally, another study comparing microscopic total resections to gross total resections showed the impact of surgical techniques on the recurrence rate of BM [75].

Prerequisites to achieving maximum safe tumor resection are intraoperative neuronavigation, neurophysiological monitoring, intraoperative imaging, and fluorescence-guided surgery. Early post-operative MRI scans are recommended to document the extent of tumor resection. The complete resection of a BM represents the crucial first step in oncological treatment, which requires further adjunct radiation and chemotherapy follow-up.

The objective of surgery is to address a central aspect of neurooncological patient care, namely the total resection of a given cerebral lesion while avoiding a neurological deficit. This key outcome represents the benchmark for both established and novel practices in most published works concerning surgical cancer therapy [36,76]. The stipulation to preserve healthy brain tissue is of utmost importance for patients, whose QoL invariably depends on their ability to ambulate, use their hands, and communicate [77,78]. Conversely, an incomplete resection puts the patient at risk of early tumor progression, which may again cause rapid functional deterioration and impairment of QoL [79,80].

A crucial predicament of neurosurgical BM resection stems from its oftentimes poor discrimination from healthy brain tissue, which renders complete resection without neurological impairment unfeasible [29,31,32]. Fluorescence-guided surgery is a rapidly developing technique in neurosurgery, having produced significant innovations in the recent decade. The most widely used neurosurgical intraoperative imaging agent is 5-aminolevulinic acid (5-ALA), which has been validated in a pivotal study by Stummer et al. in 2006 [81]. However, 5-ALA has exclusively been investigated and approved for use in patients with high-grade gliomas. At the current state, the substance has only generated mixed results in the BM population and has not entered the standard of care due to the lack of high-quality evidence from randomized controlled clinical trials [76]. In principle, these intraoperative imaging technologies are invaluable to surgical strategy; by providing a clear distinction between the lesion to be resected and surrounding healthy tissue, the surgeon is greatly aided during the most crucial process of any given tumor resection [82]. In recent investigations, fluorescein sodium has emerged as a promising agent for the intraoperative fluorescent staining of BMs [82,83].

Apart from open microsurgical tumor resection, innovative technologies for minimally invasive tumor removal are emerging. MRI-guided laser interstitial thermal therapy (MRgLITT) enables ablation of the lesion under continuous imaging control with MRI. In MRgLITT, local thermal ablation of the tumor is achieved by applying laser energy via a stereotactic-inserted laser probe. MRgLITT can be a surgical treatment option, especially for metastases that cannot be safely accessed by open resection (e.g., thalamic localization) or with contraindications for SRS or conventional RT [84,85]. The effectiveness of MRgLITT in the primary treatment of cerebral metastases has so far only been proven on the basis of retrospective studies; data from controlled studies are not yet available [86].

## 3. Definitive Radiosurgery (SRS)

The important role of SRS in the treatment of solitary BM was evaluated several decades ago in a multicenter retrospective study in North America [87]. This study included 122 patients from four institutions. The inclusion criteria exactly matched those of two randomized trials published a few years earlier that evaluated the role of surgery alone versus surgery plus WBRT [43,88]. It was a surprise to find that radiosurgery plus WBRT achieves absolutely comparable local tumor control to that achieved by surgery plus WBRT. Therefore, this study demonstrated the ablative nature of radiosurgery.

Subsequent randomized studies have shown significantly better local tumor control when WBRT is combined with radiosurgery compared to WBRT alone [89,90]. Furthermore, radiosurgery plus WBRT compared to WBRT alone even improved OS in certain subgroups of patients with favorable recursive partitioning analysis/graded prognostic assessment (RPA/GPA) [91].

Given the very good local tumor control of radiosurgery and the possible side effects of WBRT, a number of studies have attempted to answer the question of whether—in the case of 1–4 brain lesions—radiosurgery can be applied alone, without WBRT. In a large prospective randomized multicentric EORTC (European Organization for Research and Treatment of Cancer) trial, Kocher et al. [92] evaluated the role of WBRT in the treatment of patients with 1–3 BM. Four arms were considered: radiosurgery, surgery, radiosurgery plus WBRT, and surgery plus WBRT. The study showed that WBRT does not improve functional independence (the primary endpoint of the study) or OS, although it significantly reduces local and distant cerebral relapses and, moreover, the number of patients who die from neurological disease. Interestingly, local relapse after local therapy (without WBRT) was 59% after surgery alone and 31% after radiosurgery alone. In contrast to the European study, a prospective multicenter randomized Japanese study shows that in a group of patients with NSCLC and favorable prognosis (diagnosis-specific GPA 2.5–4), adding WBRT to radiosurgery (RS) can significantly improve OS [93,94]. However, in a systematic review of the Cochrane database evaluating RS alone versus RS plus WBRT, the conclusion is that WBRT has no impact on OS [95]. Therefore, radiosurgery alone remains the indicated treatment in patients with 1–4 non-resected BM.

An important argument in the decision to abandon WBRT is the significant negative effect of WBRT on cognitive function. Chang et al. [96] observed in a prospective randomized trial that patients treated with WBRT plus RS had a significant decline in learning and memory function 4 months after treatment compared to patients who were treated with radiosurgery alone. A few years later, Brown et al. reported similar results in another study, also prospective, multicenter, randomized, and cognitive function as the endpoint [97].

A Japanese multicenter prospective observational study evaluated the role of radiosurgery alone in the treatment of patients with multiple BM: 5–10 cerebral lesions with a maximum total metastatic volume of less than 15 mL [98]. In the 1194 patients with 1–10 unresectable BM included in the study, the authors find that, in terms of OS, there is no difference between patients with 2–4 metastases and those with 5–10 lesions. Patients with solitary lesions had better survival. The authors conclude that, given the side effects of WBRT, it can be abandoned in favor of radiosurgery, even if the latter should be repeated in case of relapse. Moreover, in a more recent cohort study, the authors advocated radiosurgery alone in patients with up to 15 lesions [99].

## 4. Radiation Therapy in Combination with Surgery

### 4.1. Local Adjuvant Radiotherapy of the Resection Cavity

According to international guidelines, surgical removal of BMs is recommended in patients with single, large (>3 cm) and symptomatic lesions in whom definitive radiosurgery is deemed ineligible or in rare cases that require histological confirmation [23,100]. Because 1- and 2-year LC rates following surgery were reported to range around 40% only [92,101], post-operative radiotherapy (PORT) of the surgical cavities is generally recommended.

Historically, PORT was conducted via WBRT, administering a variety of dose fractionations with single doses of 1.8–4 Gy and total doses of 20–50.4 Gy. Across several rather small studies, WBRT significantly reduced the risk of both local and distant intracerebral relapse. Also, death from neurological causes occurred less frequently after post-operative WBRT; however, there was no consistent effect on OS [25].

According to the aforementioned pivotal EORTC 22952-26001 trial, post-operative WBRT following radio-surgical treatment was not found to improve OS, but it significantly decreased QoL [92,102]. However, as reported before, post-operative WBRT did improve LC and reduce the number of neurological deaths. To make use of this local effect and simultaneously spare the healthy brain, several studies have investigated the effect of either immediate or salvage SRS instead of post-operative WBRT or watch-and-scan strategies:

Postoperative SRS instead of observation strategies did not alter OS rates but improved LC rates from 43 to 72% [101] at modest toxicity rates. Postoperative SRS instead of WBRT was superior in preserving neurocognition and QoL [6]. However, after 6 months, surgical bed control following WBRT was surprisingly significantly superior to SRS (80% vs. 87%), which may be related to challenges in target volume delineation following surgery [103]. Reserving post-operative SRS for patients with either residual or recurrent BMs following resection was analyzed in a large Japanese study and randomized against elective post-operative WBRT. The authors found comparable OS and less toxicity with salvage SRS vs. elective WBRT [104].

In conclusion, post-operative radiotherapy is still considered mandatory after surgical removal of BMs, but it should not be conducted as WBRT. Secondary to the aforementioned neuro-cognitive sequelae affecting the patient´s QoL, there is a trend toward focal radiotherapy to the resected BM site to omit or delay WBRT [25,105,106,107,108,109]. Local post-operative RT to the resection cavity spares the healthy brain and can therefore preserve neuro-cognition, and is recommended in established guidelines [6,23,24,101,110]. Normo-fractionated or modestly hypo-fractionated adjuvant RT has been investigated by several groups demonstrating satisfying control rates [100,111,112,113,114], For example, Byrne et al. applied 30–42 Gy in single-fractions of 3 Gy to the post-operative cavity in 54 patients. They achieved a local control rate of 97.0% and 88.2% at 6 and 12 months, respectively [112]. Another study demonstrated a local control rate of 83% at 1 year and 78% at 2 years in 57 patients treated postoperatively with local RT; the median dose was 48 Gy (30.0–50.4 Gy) in 25 (10–28) fractions [111]. However, most data are available for adjuvant stereotactic RT, which can be applied with single-session SRS or hypo-fractionated (3–7 treatments) stereotactic RT (hFSRT) [109,115,116,117,118,119,120,121,122]. The ideal stereotactic scheme is still under debate; cavity size and dosimetric aspects have to be taken into consideration [123]. Up to date, there are no randomized controlled trials available to determine which of the two techniques is superior in terms of efficacy (LC) and safety, namely the risk of radiation necrosis (RN). Currently, two prospective randomized trials are comparing post-operative SRS and hFSRT (NCT04114981 and NCT05160818) to answer this crucial question. Data from several predominantly retrospective studies are available that used different dose, fractionation, and contouring schemes (e.g., surgical tract included or not included, margin vs. no margin). Two trials on SRS reported 1-year LC rates of 72% and 60%, respectively [6,101]. In SRS, often no or a very small (1 mm) safety margin is applied to minimize toxic effects on the normal brain, which could in part explain the inferior LC rate. A recently published series on >500 cavities treated with stereotactic RT (SRS 15.2%, hFSRT 84.8%) demonstrated excellent LC rates of 93% with 1-year overall adverse radiation effects of 9%; in regard to the imbalanced groups, the authors could not find inferiority in SRS compared to hFSRT [124]. Another meta-analysis looking at 24 studies for definitive and post-operative RT of large BMs reported for hFSRT (405 patients, heterogeneous fractionation schemes) a LC of 87% compared to 68% in the SRS group (183 patients) [125]. Furthermore, in the majority of studies, the definitions of LC and RN were not clearly defined and depended predominantly on the institutional standards, mostly relying on radiologic findings. A recent multicenter analysis of 558 cavities treated with hFSRT (median total dose 30 Gy, median dose per fraction 6 Gy) demonstrated a 1-year LC rate of 84% and an RN rate of 4.1% [122]. In general, adjuvant hFSRT seems favorable over single-fraction radiosurgery in terms of higher LC and lower incidences of RN, most likely due to breaks between fractions and a higher biologic effective dose (BED) [122,125,126,127,128]. For hFSRT dose (BEDα/β = 10 > 48 Gy), cavity/PTV volume (<23 mL), single BM, extent of resection, and a controlled primary tumor are associated with LC [108,109,115,122,129,130,131]. Risk factors for RN are treating volume, fractional dose, and total RT dose [132,133]. Some studies described constraints for fractional (30 Gy in 5 fractions, BED10 = 48 Gy, BED2 = 120 Gy) irradiated normal brain volume receiving 20 Gy or more of 20 cm^3^ and 25 cm^3^ (V20Gy > 20 cm^3^ and V20Gy > 25 cm^3^) for the development of symptomatic RN [121,129,134,135]. RN rates for 5–7 fractions (BED2 87.5–138.1 Gy) are 0–10% with a 1-year LC of 68 to 93% [109,121,122,128,135,136]. Whereas three-fraction hFSRT (BED2 120–214.5 Gy) shows higher LC rates (88–98.9%) but a much higher incidence of RN (14–25.7%) [118,137,138,139,140]. Therefore, hFSRT with 5 to 7 fractions can be considered effective and safe.

Despite the increasing use of local adjuvant RT, there is no definite standard for target delineation. A recent consensus guideline recommended enclosing the surgical tract and, if necessary, a margin for the bone flap, venous sinus, and pia mater in cases where the BM abuts the appropriate structures [103]. Based on several studies, adding a 2–3 mm margin to the resection cavity for the planned target volume (PTV) seems beneficial for LC [116,141,142]. However, a safety margin can be discussed controversial. A meta-analysis from 2013 including 629 patients did not find an effect of an extra margin on LC, and in the aforementioned study (n = 500, SRS 15.2%, hFSRT 84.8%), no difference was detected for margin (1–3 mm; 76% of cases) vs. no margin [124,143].

Another important aspect of target volume delineation is the fact that the cavity volume can vary over time after surgery. One study on 68 cavities demonstrated, in most cases, a median percent volume change of minus 29% compared to preoperative BM volume within 1–3 days after resection, but without significant further changes over the next 6 weeks [144]. Other series stated cavity dynamics within 4 weeks after resection, and larger cavities show larger shrinkage than smaller ones [145,146,147]. This might imply a protracted start of adjuvant stereotactic RT. However, several studies have shown that a delay has a negative impact on LC [148,149]. Taken together, due to variable changes in the cavity volume within weeks after surgery, a recent MRI not older than 7 days should be obtained for RT planning [142,144,150]. Consequently, the right timing for post-surgical RT is not standardized, but besides surgical-related factors such as the patient´s performance and wound healing, the urge for a systemic treatment depending on the tumor staging should be taken into consideration [122,135,146].

Regarding the challenges of optimal target delineation and the dynamics of the resection cavity, further modalities of local RT for resectable BM are gaining increasing interest [151,152].

### 4.2. Neoadjuvant Radiation Therapy

As an alternative to adjuvant RT to the resection cavity, neo-adjuvant stereotactic radiosurgery (N-SRS) of the BM prior to resection seems to be another option. First of all, the concept of N-SRS of intact BM offers improved target volume contouring since, with adequate imaging, exact delineation of intact BM is fairly straightforward. Additional safety margins are not necessarily needed for optimal coverage, and no surgical tract has to be included in the target volume, which enables better sparing of the surrounding healthy brain. A dosimetric analysis comparing neoadjuvant RT with adjuvant hFSRT could demonstrate a significantly reduced dose exposure to the brain and favorable conformity indices [153]. Overall, irradiated target volume appears lower for pre-operative RT compared to post-operative RT, especially for smaller metastases; in addition, the adjacent brain volume receiving the prescription dose is removed during surgery, which could lead to less frequent RN and wound healing complications [154,155]. Another major aspect is that during resection, tumor cells might be disseminated, with a significant risk of leptomeningeal disease (LMD) [156,157]. Therefore, N-SRS might sterilize viable tumor cells, which are then less competent for replication when later spilled during tumor resection, potentially translating into lower rates of LMD. Also, the much-needed systemic therapy or rehabilitation could be initiated more rapidly because there is no 2–3 week delay to ensure adequate wound healing and perform adjuvant hFSRT.

Over the last few years, there has been growing evidence for the effectiveness and safety of neoadjuvant SRS for BM [158,159,160,161,162,163,164,165]. The first larger series investigated the role of N-SRS in 47 patients with 51 BM (median diameter 3.0 cm, range: 1.3–5.2 cm) undergoing resection in median within 1 day (range: 0–7 days) after N-SRS with a median dose of 14.0 Gy (range: 11.6–18 Gy) prescribed to the 80% isodose level [158]. LC was 97.8%, 85.6%, and 71.8% at 6, 12, and 24 months, respectively. Eight patients with local failure were re-operated and proved to have recurrence without RN. Local failure was more likely for lesions larger than 3.4 cm (*p* = 0.014). One trial compared pre- and post-operative stereotactic RT in 180 patients, with 189 BMs being resected. Of note, in the N-SRS group (n = 66), the marginal dose was reduced by 20% (median dose 14.5 Gy vs. 18 Gy) with no extra margin added to the gross BM volume compared to the post-surgical cohort with a safety margin of 2 mm [160]. Patients underwent BM resection within 48 h after SRS. Outcomes were similar regarding local recurrence, distant brain recurrence, and overall survival, but with significantly lower rates of symptomatic RN and leptomeningeal spread in favor of the pre-operative group, with 4.9% vs. 16.4% (*p* = 0.1) and 16.6% vs. 3.2% (*p* = 0.1) at 2 years, respectively [160]. In another study by this group, pre-operative SRS (66 patients with 71 lesions) was compared to post-operative WBRT (36 patients with 42 lesions). In analogy to the aforementioned study, the dose was reduced by 20% with no extra margin for the PTV with surgery within 48 h after neoadjuvant RT [161]. Again, results were similar for local recurrence, distant brain failure, and LMD recurrence. Rates for symptomatic RN were higher for pre-operative SRS (5.6% vs. 0.0%), and the cavity size was significantly smaller in this group [161]. Comparably, a study on 117 patients with 125 lesions treated with neoadjuvant SRS showed LC rates of 74.9% at 24 months and RN in 4.8% of cases [163]. Lately, pooled data from a multicenter cohort of 253 index lesions with N-SRS (median dose 15 Gy) were published, describing local recurrence rates of 15.0% and 17.9% at 1 and 2 years, respectively; LMD occurred in 7.6% after 24 months [162].

Overall, N-SRS followed by resection appears to be effective and safe, but there are some disadvantages. First of all, N-SRS is performed without prior histopathologic proof for BM. MRI offers a high positive predictive value for BM; however, there is still a low risk of falsely irradiating high-grade glioma, lymphoma, or even an inflammatory process with N-SRS. Furthermore, urgent surgery for space-occupying lesions should not be delayed by N-SRS. Therefore, patients with cancer of unknown primary and acute symptoms necessitating rapid decompression might not be the best candidates for N-SRS. Furthermore, more data are needed on dose and fractionation schemes as well as with regard to immunogenetic effects in interplay with checkpoint inhibitors. So far, the number of studies on N-SRS is limited compared to post-operative RT, but further prospective studies on effectiveness, safety, and QoL are underway [166,167,168].

### 4.3. Intraoperative Radiation Therapy (IORT) with Low-Energy X-rays

**Figure 1:**
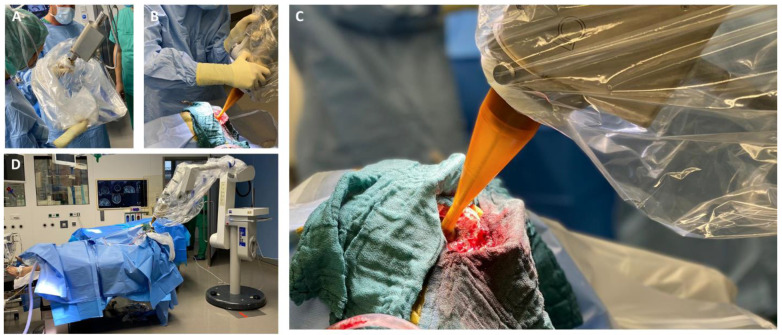
(**A**) Connection of the spherical applicator with the X-ray source and sterile draping; (**B**) Manual applicator positioning into the resection cavity; (**C**) Intraoperative view of the inserted applicator in the resection cavity; (**D**) Intraoperative set-up prior to the start of IORT.

Among the vast therapeutic arsenal for treating BM, IORT poses an interesting option in the adjuvant spectrum (Figure 1). The introduction of modern IORT for treating cancer traces back to the 1960s, when some initial reports announced promising outcomes in various anatomical locations using electron-based IORT (IOERT) [169]. Falling within the frame of brachytherapy (BT), this approach allows delivering radiation by placing an emitting source in direct contact with the target tissue during a single surgical act. The evolution of this technique over the past fifty years, since the early IOERT or BT-based IORT reports, has yielded a robust and convenient modality to still consider nowadays, despite a persistent lack of large prospective data.

#### 4.3.1. Neurosurgical Aspects in IORT

The safety of intraoperative radiation therapy (IORT) has been confirmed by an international, retrospective cohort study of 54 patients [170]. A more recent publication assessed the relationship between IORT and surgical complications, finding no substantial differences [171]. Furthermore, a case report highlighted no additional adverse events when delivering IORT during awake craniotomies [172]. In addition, a safety analysis addressing the neurological functionality of 70 patients found no significant IORT-associated detriment after surgery [173].

However, the following surgical aspects should be considered to guarantee a safe and effective IORT: (i) cortical to subcortical location and sufficient width of corticotomy; (ii) location of metastasis is favorable for safe applicator insertion and positioning (preferably non-eloquent areas); (iii) the required distance to organs at risk should be confirmed intraoperatively by using the neuronavigation; and (iv) a dry resection cavity should be prepared to optimize radiation kinetics (no active effusion of CSF) [171,174]. IORT can be used independently of the primary tumor; however, pathological examination of a fresh frozen section throughout surgery should be undertaken, especially in cases with an unknown primary.

#### 4.3.2. Radiotherapeutic Aspects in IORT

A publication from the 1990s described the outcomes of patients receiving IOERT as an adjuvant approach. An approximately 84% 1-year LC rate (LCR) was observed in 43 resected BMs, comparable to those reported by modern large randomized trials [6,101,175]. A similar experience with isotope-based intraoperative brachytherapy (IBT), assessed prospectively using a Cs131 source, resulted in no local recurrences or RN after 1.5 years in 42 patients carrying 46 BM [176]. Likewise, a single-center, retrospective study evaluating stereotactically placed I125 seeds as a minimally invasive alternative for non-resectable BM demonstrated low 1-year local recurrence rates (5.4%) and no RN in 77 patients [177]. This approach eases obtaining histopathological samples and confirming the nature of the entity to treat within the same surgical act [178].

Kilovoltage (low-energy X-rays) IORT entered the scene as early as 1907, with a first publication on abdominal malignancies [179]; nonetheless, it has fallen into a prolonged scientific silence since then. Recent advances in the field, such as miniaturized portable accelerators, have enabled interesting output features (e.g., 50 kV) and re-opened a window for further enhancing a personalized and targeted approach. A sharp fall-off dose profile, inherent to these devices, assures a radially homogeneous dose distribution while reducing organs at risk (OARs) exposure [180]. This particular characteristic is of great interest to neuro-oncologists when it comes to BM treatment.

**Figure 2:**
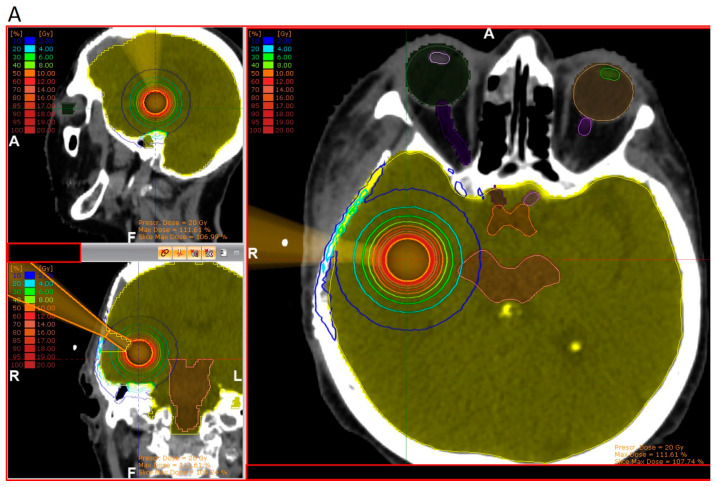

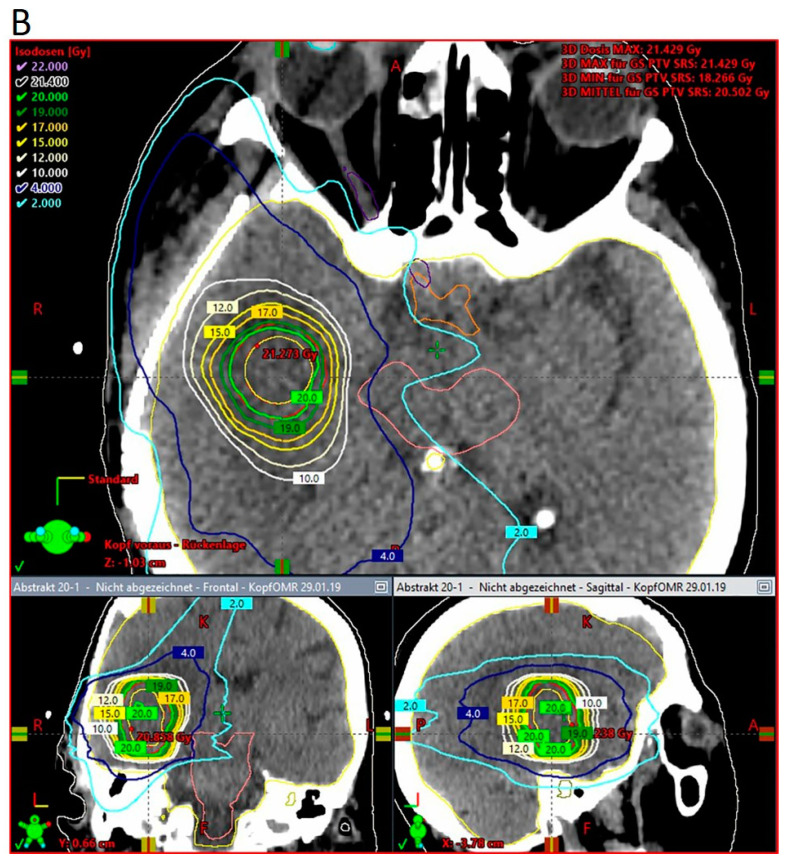
Three-dimensional view of dose distribution profiles, comparing (**A**) IORT and (**B**) SRS for the same target. Reprinted with permission from Sarria et al. [181]. Copyright year 2021, copyright owner’s name Sarria et al. Under the terms of the Creative Commons Attribution License (CC BY) [181].

According to pathology-based reports on tissue infiltration surrounding BM, a clinical target between 1 and 4 mm should be considered in the radiotherapy adjuvant setting, depending on the primary histology [33,35]. With approximately 80% of the prescribed surface dose at 3 mm from the surface, kV IORT provides an outstanding treatment outline while avoiding the usually large volumes required during adjuvant SRS. Subsequent dosimetric comparisons between IORT and SRS as dose-escalation strategies for resected glioblastoma and BM, respectively, demonstrated up to three times lower brain V12G (volume of brain receiving 12 Gy) results, favoring IORT [181,182]. An exemplary dosimetric comparison between both modalities is displayed in Figure 2 [181].

Current retrospective studies have explored the initial clinical outcomes of these patients (Table 1). An earlier publication depicted the initial results of a seven-patient collective, finding a 1-year LCR of 86% [182]. An additional dosimetric comparison against SRS in this same cohort suggested standardizing 30 Gy for further interventions [182]. A German report on 40 patients carrying 44 BM, treated with kV IORT by means of a spherical applicator and a median dose of 20 Gy, found an 84.3% 1-year LCR and a 2.5% RN rate [183]. A second publication, resulting from an international pooled cohort, showed a 1-year 88% LCR in 54 patients with a 7% RN rate [170]. A newly published work adds up to suggesting encouraging safety and control outcomes, similar to all the above-mentioned [184].

It is noteworthy that the reported 12 to 23 min of additional surgical time could help save patient and professional time and reduce hospital visits related to planning and adjuvant treatment delivery. The advantage of treatment consolidation makes IORT particularly interesting for patients undergoing resection of solitary metastasis, anxious patients, or patients with limited compliance for standard post-operative radiation. In cases with a high disease burden, IORT may guarantee a timely start of oncological therapy. Furthermore, some publications have highlighted the role of IORT in the activation of an immune anti-tumoral response [185,186,187]. Certain radiobiological plausibility could be inferred in the BM setting, as this could play a central role in systemic disease control. Of course, this conception is preliminary, and clinical validation is required.

Given the aforementioned preliminary evidence and rationale, prospective studies (NCT03226483, NCT04690348), an international BM kV-IORT registry, and a recently announced phase-III trial will elucidate the role of this approach in the management of BM. In addition, ongoing research will determine the benefit of image-guided IORT (IG-IORT), which allows real-time planning and positioning correction, as well as the dosimetric impact of employing hemostats. The indications/contraindication and advantages/disadvantages of the different RT modalities in combination with surgery are summarized in Table 2.

### 4.4. Re-Irradiation of Recurrent Brain Metastases

The treatment options of immunotherapy and targeted therapy led to the prolonged survival of many stage IV cancer patients. This intensified the focus on optimal treatment strategies for patients with recurrent BM (rBM) after initial RT. So far, published data comprises only retrospective, mostly monocentric case series, and recommendations from guidelines are lacking as well. From a radiation oncological perspective, it is appropriate to distinguish between patients with recurrences after WBRT and patients with local failures after focal radiotherapy.

Despite missing prospective data, SRS is the treatment of choice for oligometastatic recurrences (1–4 metastases) after WBRT due to its high efficiency and minimal toxicity [188,189]. The focus of the published data lies in appropriate patient selection (Supplementary Table S1). KPS, the interval from WBRT, and controlled systemic disease are common selection criteria in this situation. As RN is rare after WBRT without focal RT, there is only a limited diagnostic dilemma in this situation.

The situation of rBM after focal RT is definitively more challenging due to the limited sensitivity and specificity of available non-invasive diagnostic procedures to distinguish it from RN [190,191,192,193]. As combinations of RN and rBM occur, even a biopsy may be subject to sample error. This emphasizes the role of salvage surgery in this setting [190]. After surgery, focal re-radiation of the cavity increases LC [194]. Focal RT of suspected rBM holds the risk of unintentional treatment of RN with possible aggravation of neurologic symptoms. Thus, several published series show favorable LC rates with only moderately increased RN rates (Supplementary Table S2). Despite increasing numbers of patients with suspected rBM (versus RN), the optimal diagnostic workup in this situation remains unclear [195,196].

## 5. Conclusions

To date, the best radio-oncologic procedure for resected BM is still under debate. In general, for low numbers of BM, WBRT is deferred in favor of local RT. Although local stereotactic RT to the resection cavity is performed in most centers, neoadjuvant RT and IORT are moving into greater focus based on convincing clinical data. Patient-centered decision-making based on the tumor volume prior to surgery, presence of symptoms, location, post-operative cavity volume, systemic treatment options, number of BM, availability of systemic treatment options, and/or extracranial tumor status will lead clinicians to the most appropriate treatment mode. Overall, IORT and post-operative cavity RT seem to be fully comparable and therefore present valid treatment alternatives.

## Figures and Tables

**Table 1:** Studies investigating clinical outcome after IORT in BM.

Reference	Pat.	Median Dose	1-Year LCR	1-Year DBC	1-Year OS	Median OS	RN	LMD
	(n)	(Gy)	(%)	(%)	(%)	Months	(%)	(%)
Cifarelli, 2019 [170]	54	30 (surface)	88	58	73	NR	7	3
Diehl, 2022 [184]	18	20 (surface)	92.9	71.4	58	22.8	11.1	5.5
Kahl, 2021 [183]	40	20 (surface)	84.3	33.50	61.6	26.4	2.5	10
Vargo, 2018 [182]	7	30 (surface)	86 *	NR	86 *	NR	0 *	NR
Weil, 2015 [174]	23	14 (2 mm)	NR	NR	NR	30	NR	NR

Abbreviations: LCR: local control rate, DBC: distant brain control, OS: overall survival, RN: radionecrosis, LMD: leptomeningeal dissemination, NR: not reported. * LCR, OS and RN after 6.2 months.

**Table 2:** Overview of RT modalities in combination with surgery for resectable brain metastases.

	Indication	Contraindication	Advantage	Disadvantage
Postoperative Whole Brain Radiation Therapy (WBRT)	High tumor burden Meningeosis carcinomatosa	Obsolete for low number of brain metastases (≤4) and good clinical state	Good loco-regional control Low rates of leptomeningeal disease Radiation necrosis is very rare	Risk for cognitive decline Longer post-operative treatment course
Local stereotactic RT of the resection cavity Linear accelerator (IMRT/VMAT ^#^) Gamma Knife Cyber Knife	Standard of care	High tumor burden Meningeosis carcinomatosa	Good local control Low rates of radiation necrosis	Protraction of adjuvant systemic treatment PTV delineation can be challenging depending on size and configuration of cavity
Neoadjuvant Radiotherapy (prior to resection)	Yet, no recommendation according to current guidelines ^‡^	Urgent indication for resection/decompression Cancer of unknown primary High tumor burden Meningeosis carcinomatosa	Straightforward PTV delineation Potentially better sparing of non-affected brain compared to local adjuvant stereotactic RT Accelerating of post-operative period with earlier start of systemic treatment/rehabilitation and better patient convenience	Unclear effect on (molecular-) pathologic work-up of tissue No standard for dose and fractionation
Intra-operative low-energy RT (IORT)	Yet, no recommendation according to current guidelines ^‡^	IORT technically not feasible (e.g., short distance to organs at risk, excessive fluid egress from opened ventricle) High tumor burden Meningeosis carcinomatosa	favorable dosimetry with high radiobiologic effectiveness in depth of 1–3 mm Steep dose gradient Low rates of radiation necrosis Accelerating of post-operative period with earlier start of systemic treatment/rehabilitation, and better patient convenience	Pre-/post-operative SRS needed for further non-resected brain metastases

^#^ IMRT: Intensity modulated radiotherapy, VMAT: Volumetric arc RT. ^‡^ EANO-ESMO/ASCO-SNO-ASTRO EANO: European Association for Neuro-Oncology, ESMO: European Society for Medical Oncology, ASCO: American Society for Medical Oncology, SNO: Society for Neuro-Oncology, ASTRO: American Society for Radiation Oncology).

## Supplementary Materials

The following supporting information can be downloaded at: https://www.mdpi.com/article/10.3390/cancers15143670/s1, Table S1: Retrospective data on re-irradiation of resected brain metastases after whole brain radiation therapy; Table S2: Retrospective data on re-irradiation of resected brain metastases after focal radiotherapy. References [197,198,199,200,201,202,203,204,205,206,207,208,209,210,211,212,213,214,215] are cited in the Supplementary Materials.

## Abbreviations

5-ALA	5-aminolevulinic acid
ALK	anaplastic lymphoma-kinase
ASCO	American Society of Clinical Oncology
ASTRO	American society for radiation oncology
BM	brain metastases
CRC	colorectal cancer
CSF	cerebrospinal fluid
EANO	European Association of Neuro-Oncology
EGFR	epidermal growth factor receptor
EORTC	European Organization for Research and Treatment of Cancer
ESMO	European Society for Medical Oncology
GPA	graded prognostic assessment
HER+	human epidermal growth factor receptor positive
hFSRT	hypo-fractionated stereotactic radiotherapy
IBT	intraoperative brachytherapy
ICP	intracranial pressure
IG-IORT	image-guided IORT
IOERT	intraoperative electron radiotherapy
IORT	intraoperative radiotherapy
KPS	Karnofsky performance status
LC	local control
LCR	local control rate
LITT	laser interstitial thermal therapy
LMD	leptomeningeal disease
MRgLITT	MRI-guided laser interstitial thermal therapy
MRI	magnetic resonance imaging
NSCLC	non-small-cell lung cancer
N-SRS	neoadjuvant stereotactic radiosurgery
OAR	organs at risk
OS	Overall Survival
PFS	progression-free survival
PORT	post-operative radiotherapy
PTV	planned target volume
QoL	quality of life
rBM	recurrent brain metastases
RCC	renal cell carcinoma
RN	radiation necrosis
RPA	recursive partitioning analysis
RT	radiotherapy
SCLC	small-cell lung cancer
SNO	Society for Neuro-Oncology
SRS	stereotactic radiosurgery
TNBC	triple-negative breast cancer
V12G	brain volume receiving 12 Gy
V20G	brain volume receiving 20 Gy
WBRT	whole-brain radiation therapy
