# Supplementary material for: Opportunities and Alternatives of Modern Radiation Oncology and Surgery for the Management of Resectable Brain Metastases
Source: Cancers (Basel). 2023 Jul 19;15(14):3670. doi: 10.3390/cancers15143670 (PMC10377800; doi:10.3390/cancers15143670)
Supplement: Supplementary file 1 [file cancers-15-03670-s001.zip › cancers-2379723-supplementary.pdf]

Supplementary Table S1. Retrospective data on re-irradiation of resected brain metastases after whole brain radiation therapy.

**Author**	**Pat.** (n)	**Met.** (n)	**Marginal SRS dose** (Gy)	**Median PTV** (cm^3)	**Median interval from WBRT** (months)	**Median OS after Re RT** (months)	**Median interval to progression after Re RT** (months)	**1-year LC** (%)	**Prognostic factors**	**Severe toxicity**
Caballero, 2012 [197]	310	3 (median)	17-18.5	5.8	8.1	8.4	NR	NR	differ from primary tumor	NR
Chao, 2008 [198]	111	NR	RTOG 90-05 protocol	NR	NR	9.9	5.2	NR	Max dose < 22Gy and tumor > 2cm were predictive for local failure	RN: 2 pat.
Huang, 2013 [199]	56	4 (median)	17	NR	10.5	10.8	6.5	NR	high KPS + control of systemic tumor	NR
Maranzano, 2012 [200]	69	NR	20	NR	11	10	NR	74 ± 4	KPS, neuro functional tests	6% RN (asympt.)
Noel, 2001 [201]	54	NR	14.4 (median)	NR	9	NR	NR	91.3	RPA class, SIR Score, interval from WBRT	Headache: 2 pat. alopecia: 2 pat.
Olson, 2012 [202]	27	NR	15-24	2.7	8	3	3.5	76.5	none	NR
Yomo, 2013 [203]	77	NR	20	8.1	10.6	8.2	NR	76.6	KPS + systemic tumor control, primary tumor, PTV size	NR

Abbreviations: Pat.: patients, Met.: metastases, SRS: stereotactic radiosurgery, WBRT: whole brain radiation therapy, OS: overall survival, RT: radiotherapy, LC: local control, NR: not reported, RTOG: radiation therapy oncology group, RN: radiation necrosis, KPS: Karnofsky performance status, RPA: recursive partitioning analysis, SIR: standardized infection ratio, PTV: planned target volume

Supplementary Table S2. Retrospective data on re-irradiation of resected brain metastases after focal radiotherapy.

Author	Pat. (n)	Met. (n)	Median Dose of 1st SRS (Gy)	Median PTV of 1st SRS (cm^3)	Median Dose of 2nd SRS (Gy)	Median PTV of 2nd SRS (cm^3)	Median interval 1st-2nd SRS (months)	Median OS after Re SRS (months)	1-year OS (%)	1-year PFS after Re RT (%)	1-year LC (%)	Prognostic factors	Severe toxicity
Balermpas, 2018 [204]	31	32	18	2	19	2.5	16.5	NR	61.7	38.7	79.5	none	16.1% RN, 12.9% grade 3 tox
Holt, 2015 [205]	13	15	21	4.3	21 in 3 fx	9.4	6.4	9	43.1	40.4	75	none	13% RN (sympt)
Iorio-Morin, 2019 [206]	56	75	20	0.86	18	1.19	11	14	56.1	NR	68	none	5% RN 8.3% edema
Jayachandran, 2014 [207]	19	27	22	1.29	17.3	6.6	NR	26	NR	NR	75	none	21% RN
Kim, 2013 [208]	32	71	NR	NR	17	NR	6.4	7.9	NR	NR	77 (6 months)	none	3 RN (sympt)
Koffer, 2017 [209]	22	24	18	2.25	15.5	3.3	13.4	8.78	37.5	NR	61	PTV >4cm^3 => reduced LC	16.7% RN
Kowalchuk, 2022 [210]	102	123	NR	NR	18	NR	12	NR	NR	NR	79	V12 >9 cm^2 + Dmax > 40Gy => sympt. RN	20% RN (7% sympt)
Terakedis, 2014 [211]	37	43	18	NR	18	NR	9	8.3	NR	NR	80.6	none	16% RN
Rana, 2017 [1212]	28	32	24	0.48 (GTV)	26.5	1.35 (GTV)	9.7	22	90	NR	88	none	18% RN (sympt)
McKay, 2017 [213]	32	46	20	1.28	20	0.98	19	N R	70	NR	79	cum. V40 predict. f. RN (0,28cm^3 10% 0,76cm^3 20% 1,6cm^3 50%)	30% RN 24% RN (sympt)
Minniti, 2016 [214]	43	47	NR	NR	21-24 in 3 fx	16.4	17	10	37	NR	70	KPS+ controlled systemic disease	19% RN (radiol)
Moreau, 2018 [215]	30	36	NR	1.48	18	4.8	>10	14,2	65	NR	67.8	PTV> 3cm^3 => worse LC PTV> 7cm^3 => increased tox.	10% RN (radiol), 13% bleeding, 10% edema

Abbreviations: Pat.: patients, Met.: metastases, SRS: stereotactic radiosurgery, PTV: planned target volume, OS: overall survival, PFS: progression-free survival, RT: radiotherapy, LC: local control, NR: not reported, RN: radiation necrosis, tox.: toxicity, fx: fractions, V12: brain volume receiving 12 Gy, Dmax: maximal dose, GTV: gross tumor volume, cum. V40: cumulative brain volume receiving 40 Gy, KPS: Karnofsky performance status
